# Distinct kinetics of inhibitory currents in thalamocortical neurons that arise from dendritic or axonal origin

**DOI:** 10.1371/journal.pone.0189690

**Published:** 2017-12-18

**Authors:** Sunggu Yang, Gubbi Govindaiah, Sang-Hun Lee, Sungchil Yang, Charles L. Cox

**Affiliations:** 1 Department of Nano-bioengineering, Incheon National University, Incheon, Korea; 2 Department of Molecular & Integrative Physiology, University of Illinois, Urbana, Illinois, United States of America; 3 Department of Neurology, University of Arkansas for Medical Sciences, Little Rock, Arkansas, United States of America; 4 Department of Biomedical Sciences, City University of Hong Kong, Tat Chee Avenue, Kowloon, Hong Kong; 5 Department of Physiology, Michigan State University, East Lansing, Michigan, United States of America; McLean Hospital/ Harvard Medical School, UNITED STATES

## Abstract

Thalamocortical neurons in the dorsal lateral geniculate nucleus (dLGN) transfer visual information from retina to primary visual cortex. This information is modulated by inhibitory input arising from local interneurons and thalamic reticular nucleus (TRN) neurons, leading to alterations of receptive field properties of thalamocortical neurons. Local GABAergic interneurons provide two distinct synaptic outputs: axonal (F1 terminals) and dendritic (F2 terminals) onto dLGN thalamocortical neurons. By contrast, TRN neurons provide only axonal output (F1 terminals) onto dLGN thalamocortical neurons. It is unclear if GABA_A_ receptor-mediated currents originating from F1 and F2 terminals have different characteristics. In the present study, we examined multiple characteristics (rise time, slope, halfwidth and decay τ) of GABA_A_ receptor-mediated miniature inhibitory postsynaptic synaptic currents (mIPSCs) originating from F1 and F2 terminals. The mIPSCs arising from F2 terminals showed slower kinetics relative to those from F1 terminals. Such differential kinetics of GABA_A_R-mediated responses could be an important role in temporal coding of visual signals.

## Introduction

In the dorsal lateral geniculate nucleus (dLGN), the primary visual input from retina constitutes a relatively small fraction (~10%) of their synaptic inputs onto thalamocortical neurons [1, 2]. By contrast, the vast majority of afferent synaptic inputs onto thalamocortical neurons consist of inhibitory, corticothalamic, and brainstem inputs. Inhibitory inputs to thalamocortical neurons primarily arise from thalamic reticular nucleus (TRN) neurons and local dLGN interneurons. The local interneurons are activated by retinogeniculate afferents, providing feed-forward inhibition to thalamocortical neurons, and these inputs play a significant role in temporal precision and receptive field properties of thalamocortical neurons [3–6]. TRN neurons are situated to provide feedback inhibition in the thalamocortical pathway and feedforward inhibition in the corticothalamic pathway [7, 8]. In addition, the reciprocal connectivity between thalamocortical neurons and TRN neurons serve as the underpinnings for intrathalamic oscillations that occur during certain sleep states and pathophysiological conditions such as absence epilepsy [9–14].

The dLGN interneurons are unique and interesting in that they give rise to two distinct types of output onto thalamocortical neurons: classical axonal outputs via F1 terminals and presynaptic dendrites (named F2 terminals) [15–21]. Retinal inputs onto thalamocortical neurons provide monosynaptic excitation via a glutamatergic retinogeniculate synapse and disynaptic inhibition via the F2 terminal (presynaptic dendrite of interneuron). These two synapses onto the thalamocortical neurons are within very close proximity, and form a *triadic* structure [2, 22]. Our previous studies have shown that the activation of metabotropic glutamate receptors (mGluRs), which are located on the dendrites of interneurons, leads to GABA release from F2 terminals resulting in lasting inhibition of thalamocortical neurons [19, 23, 24].

Although previous electrophysiological studies have shown the presence of both F1- and F2-terminal mediated inhibition onto thalamocortical neurons, it is unclear if ISPCs arising from these different origins have distinct characteristics. Anatomical studies indicated differential distribution of F1 and F2 terminals on thalamocortical neurons: F2 terminals tend to be present on proximal dendrites, whereas F1 terminals are found on proximal and distal dendrites [2, 22, 25]. In the present study, we have systematically examined IPSC kinetics originating from presumed F1 and F2 terminals. The identified differences in the kinetics of inhibitory responses arising from F1 and F2 terminals could impact the temporal precision of sensory information transfer to the neocortex.

## Materials and methods

### Brain slice preparation

Sprague-Dawley rats (postnatal age: 10–16 days, males and females) were deeply anesthetized with sodium pentobarbital (55 mg/kg), the brains were quickly removed, and placed into chilled (4°C), oxygenated (5% CO_2_/95% O_2_) slicing solution containing (in mM): 2.5 KCl, 1.25 NaH_2_PO_4_, 10.0 MgSO_4_, 0.5 CaCl_2_, 26.0 NaHCO_3_, 11.0 glucose, and 234.0 sucrose. Slices (300 μμ thickness) were cut using a vibrating tissue slicer in the coronal plane for dLGN recordings and in the horizontal plane for ventrobasal nucleus (VB) recordings. Slices were then transferred to a holding chamber containing oxygenated physiological saline that contained (in mM): 126.0 NaCl, 2.5 KCl, 1.25 NaH_2_PO_4_, 2.0 MgCl_2_, 2.0 CaCl_2_, 26.0 NaHCO_3_, and 10.0 glucose. Individual slices were transferred to a recording chamber maintained at 32°C, and oxygenated physiological saline was continuously superfused at a rate of 2.0 ml/min. All procedures performed were approved by the IACUC at the University of Illinois, Urbana-Champaign.

### Whole-cell recording procedures

Recordings were obtained using the whole-cell configuration as previously described [26]. Recording pipettes had tip resistances of 3–7 MΩ when filled with an intracellular solution containing (in mM): 117.0 Cs-gluconate, 13.0 CsCl, 1.0 MgCl_2_, 0.07 CaCl_2_, 0.1 EGTA, 10.0 HEPES, 2.0 Na_2_-ATP, 0.4 Na-GTP, and 0.3% biocytin. For paired recordings, the Cs^+^-based internal solution was replaced with K^+^-based internal solution. The pH and osmolarity of intracellular solution was adjusted to 7.3 and 290 mOsm, respectively. The internal solution resulted in ~10 mV junction potential that was corrected in the voltage measures. A fixed-stage microscope (Axioskop2, Carl Zeiss, Inc.) equipped with differential interference contrast optics and a 63× water-immersion objective was used to view individual neurons within the slice. Membrane voltages and currents were recorded using a Multiclamp 700B amplifier (Molecular Devices, Sunnyvale, CA). Inhibitory postsynaptic currents (IPSCs) were recorded at holding potential of 0 mV using Cs^+^-based internal solution. Only neurons with stable access resistances <15 MΩ were included for analysis in this study. Interneurons and thalamocortical neurons were distinguished by both physiological and morphological criteria. Relay neurons produced a stereotypical voltage-dependent burst firing response. In contrast, interneurons had a more pronounced depolarizing sag in response to hyperpolarizing current pulses than thalamocortical neurons. Interneurons had a bipolar appearance with small, fusiform somata with dendrites emanating from the opposite poles of the soma, whereas thalamocortical neurons typically had an oval soma with four to seven processes projecting from the soma [24].

Concentrated stock solutions of pharmacological agents were initially prepared and stored as recommended by manufacturers. The stock solution was diluted in physiological saline to a final concentration just prior to use. All compounds were purchased from Tocris (Ellisvile, MO) or Sigma (St. Louis, MO).

### Data acquisition and analyses

Spontaneous synaptic events were digitized, stored and analyzed off-line using pCLAMP software (Molecular Devices, Sunnyvale, CA) and Mini Analysis (Synaptosoft, Fort Lee, NJ). The detection of miniature ISPCs (mIPSCs) was accomplished by setting a threshold at two times greater than the baseline level in presence of the GABA_A_ antagonist, SR95531. mIPSC amplitudes, rise times (10–90% amplitude), rising slopes, half-width, and decay time constant (τ) were calculated and compared across different experimental groups. Multipeak mIPSCs, extremely large amplitude (>100 pA), and slow rise time (>5 ms) events were excluded from analyses. Statistical analyses consisted of a student’s *t* test and Fishers posthoc test, as noted. Data are presented as mean ± SD.

## Results

**Fig 1:**
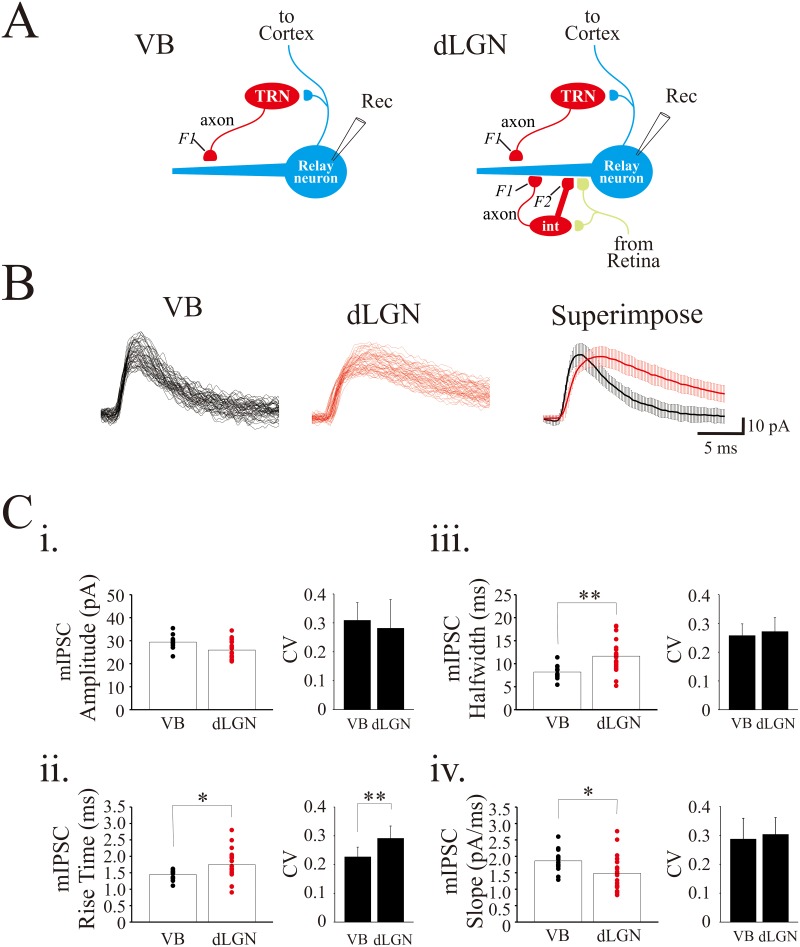
The kinetics of mIPSCs differs in VB and dLGN thalamocortical neurons. **A**. Schematic diagram illustrating VB which lacks interneurons and receives F1 terminal inputs from TRN. By contrast, dLGN thalamocortical neurons receive both F1 and F2 terminal inputs from interneurons and F1 inputs from TRN. **B**. Superimposed current traces of 50 consecutive mIPSCs recorded in dLGN (*left*) and VB (*middle*) thalamocortical neurons. Superimpose of the averaged mIPSCs (± SD) from VB and dLGN thalamocortical neurons (*right*). **C**. Population data illustrating amplitude (i), rise time (ii), halfwidth (iii), and slope (iv) from VB and dLGN thalamocortical neurons. * p<0.05, ** p<0.01.

As illustrated in Fig 1, thalamocortical neurons in VB only receive F1 terminal inputs from TRN neurons due to the lack of local circuit interneurons. By contrast, dLGN thalamocortical neurons receive F1 terminal innervation from local interneurons and TRN neurons, and F2 terminal inputs from local interneurons [18]. mIPSCs were recorded from dLGN (n = 21) and VB (n = 13) thalamocortical neurons in the presence of tetrodotoxin (TTX, 1μM) (Fig 1B). The mIPSCs recorded from dLGN neurons had significantly greater rise times (1.7 ± 0.5 ms, n = 21) and wider halfwidths (11.6 ± 3.5 ms) than those recorded from VB neurons (rise time: 1.4 ± 0.2 ms; n = 13; p < 0.05, t-test; halfwidth: 8.19 ± 1.46; p < 0.01, t-test). Meanwhile, the mIPSC decay slope was significantly slower in dLGN (14.8 ± 5.2 pA/ms) than VB neurons (18.6 ± 3.6 pA/ms; p < 0.05, t-test). mIPSC amplitude did not differ between dLGN and VB neurons (dLGN: 29.4 ± 0.8 pA; VB: 25.9 ± 0.89 pA, p > 0.05, t-test). The rise time of mIPSCs recorded in dLGN relay neurons had larger coefficient of variation (CV), an index of variability (CV: 0.29 ± 0.04), than that in VB neurons (CV: 0.23 ± 0.03). We hypothesized that large variation in rise times of dLGN mIPSCs could be due to mixture of F1- and F2-mediated IPSCs in dLGN neurons.

**Fig 2:**
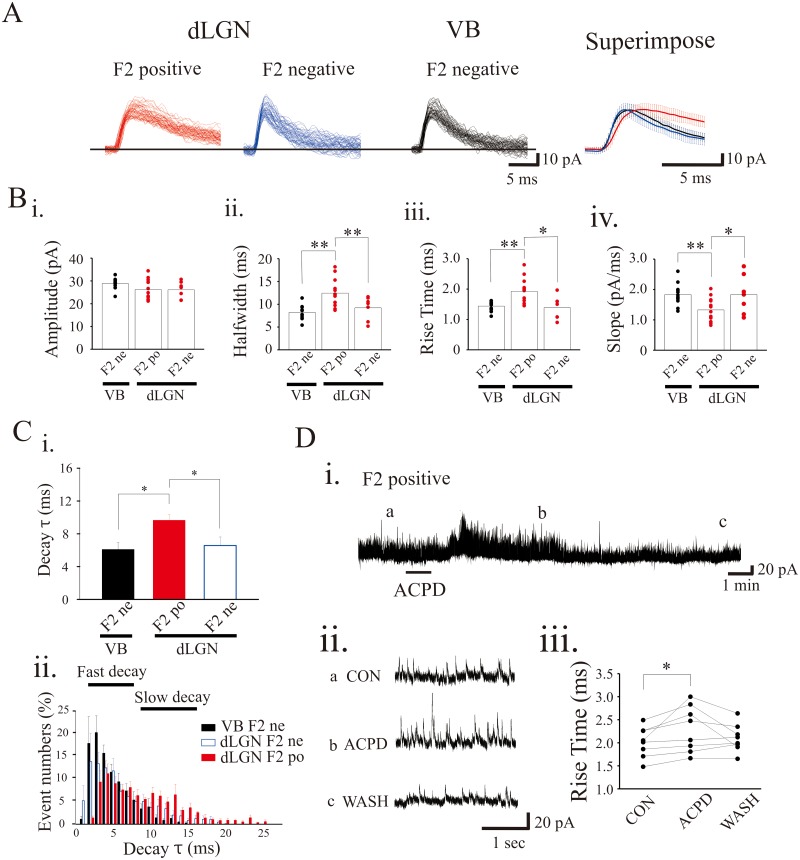
Slow mIPSC kinetics of F2 terminals. **A**. Representative current traces revealing individual mIPSCs: F2-positive dLGN: subpopulation of dLGN neurons that have a TTX-insensitive increase in mIPSCs by ACPD. F2-negative: dLGN neurons that do not show TTX-insensitive increase in mIPSC activity to ACPD, and VB: neurons that do not show TTX-insensitive increase in mIPSC activity to ACPD. The superimpose is the averaged mIPSCs for each of the 3 representative types of neurons. **B**. Summary plots of the population data showing amplitude (i), rise time (ii), halfwidth (iii), and slope (iv) of mIPSCs. **C**. The dLGN F2-positive neurons had the longest decay τ among three groups. The analysis of event numbers over the degree of decay τ showed the bimodal distribution of mIPSC in dLGN F2-positive neurons. **D**. Short-term (15 s) exposure to ACPD increases mIPSC frequency via F2 outputs. The representative current trace showing the mIPSCs before ACPD (iia), after ACPD (iib) and following wash (iic). iii. ACPD significantly increases the mIPSC rise time compared to pre-drug condition. Error bars represent mean ± SD. * p<0.05, ** p<0.01.

In our earlier studies, in a subset of dLGN thalamocortical neurons the activation of mGluRs in the presence of TTX led to a selective increase in F2 terminal outputs (F2-positive neurons), thereby leading to an increase in GABA-mediated IPSCs in thalamocortical neurons [19, 23, 24, 27, 28]. By contrast, in VB neurons and a subset of dLGN neurons, mGluR agonists applied in the presence of TTX did not alter mIPSC activity (F2-negative neurons; Fig 2A and 2C). We analyzed various mIPSC kinetics from these three different groups of neurons: F2-positive dLGN (n = 13), F2-negative dLGN (n = 7), and VB (n = 12) neurons. The mIPSC amplitude did not statistically differ between these three neuron groups (Fig 2Bi, Table 1). The mIPSCs of F2-positive dLGN neurons had significantly slower rise times and greater halfwidths in comparison to dLGN F2-negative neurons and VB neurons (Fig 2Bii and 2Biii; Table 1). In addition, the decay slope of mIPSCs in F2-positive dLGN neurons was significantly less than those in dLGN F2-negative neurons and VB neurons (Fig 2Biv, Table 1). In contrast, there were no significant differences between the rise time, halfwidth, and decay slope of dLGN F1-negative neurons and VB neurons (Table 1).

**Table 1:** The characteristics of mIPSCs from dLGN and VB thalamocortical neurons.

	LGN F2 positive (n = 13)	LGN F2 negative (n = 7)	VB (n = 12)	Posthoc Fisher LSD
Amplitude (mV)	26.2 ± 4.4	26.1 ± 3.7	28.9 ± 2.4	*F2+ vs. F2*-: p = 0.95, ns *F2+ vs. VB*: p = 0.25, ns *F2- vs. VB*: p = 0.22, ns
Rise Time (ms)	1.9 ± 0.4	1.4 ± 0.4	1.4 ± 0.2	*F2+ vs. F2*-: p = 0.0016, * F2+ vs. VB: p = 0.0009, * F2- vs. VB: p = 0.75, ns
Halfwidth (ms)	12.9 ± 3.3	9.2 ± 2.6	8.2 ± 1.5	*F2+ vs. F2*-: p = 0.0087, * F2+ vs. VB: p = 0.002, * F2- vs. VB: p = 0.41, ns
Slope (pA/ms)	13.1 ± 3.7	18.6 ± 6.2	18.6 ± 3.6	*F2+ vs. F2*-: p = 0.02, * F2+ vs. VB: p = 0.0073, * F2- vs. VB: p = 0.99, ns
Decay τ (ms)	9.7 ± 0.7	6.6 ± 1.1	6.1 ± 0.8	F2+ vs. F2-: p = 0.0087, * F2+ vs. VB: p = 0.002, * F2- vs. VB: p = 0.57, ns

Considering the halfwidth of the mIPSCs differed between two different neurons, we subsequently calculated the decay time constant (τ) of the mIPSCS. The mIPSCs of F2-positive dLGN neurons had significantly longer τ compared to those in F2-negative dLGN neurons and VB neurons (Fig 2Ci, Table 1). As illustrated in Fig 2Cii, τ is bimodally distributed in dLGN F2-positive neurons, but not in dLGN F2-negative and TRN neurons, suggesting diversity of mIPSCs in dLGN neurons. The bimodal distribution in the F2-positive dLGN neurons suggests multiple populations that could correspond to F1 and F2 terminal innervation. In F2-positive dLGN neurons, subsequent exposure to ACPD (25–100 μM) produced a robust increase in mIPSC activity. We analyzed the kinetics of mIPSCs pre- and post-ACPD in these neurons. The average rise time in pre-ACPD (2.02 ± 0.33 ms) significantly increased after ACPD application (Fig 2D, 2.30 ± 0.50 ms, n = 8; p < 0.05, paired t-test). These data were consistent with our early finding that the F2 activation showed slow kinetics of mIPSCs.

**Fig 3:**
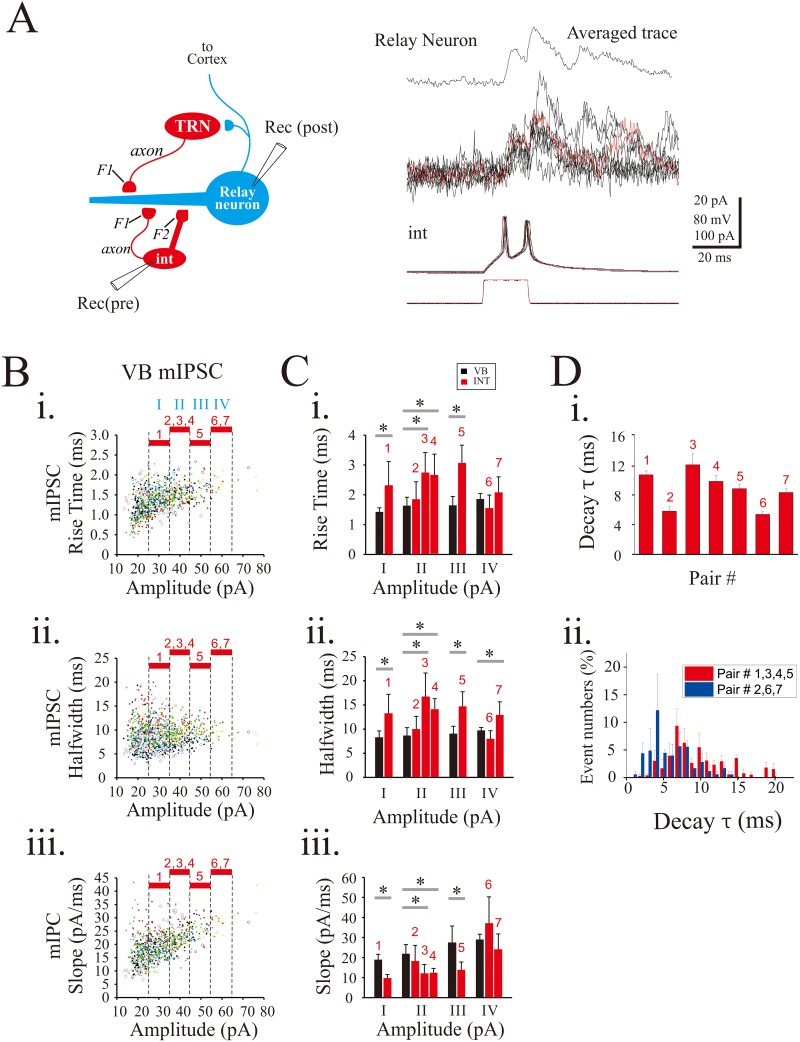
Diverse IPSC kinetics originating from local interneurons. **A**. Schematic diagram illustrating dual whole-cell recordings from synaptically-coupled dLGN interneurons and thalamocortical neurons (*left*) and electrophysiological responses to depolarizing current (*right*). Current-induced excitation of interneurons elicited two sequential ueIPSCs in dLGN thalamocortical neurons. **B**. Population data illustrating rise time (i), halfwidth (ii), and slope (iii) as a function of mIPSC amplitude obtained from VB neurons (n = 13). Note the number on the top indicating the same amplitude comparison of local interneuron ueIPSC. **C**. Population data illustrating rise time (i), halfwidth (ii), and slope (iii) as a function of amplitude obtained from VB mIPSCs and local interneuron ueIPSC (red). **D**. The activation of local interneurons induces GABA release in two modes, fast decay τ (pair #2, #6, #7) and slow decay τ (pair #1,#3, #4, #5). *, p<0.05.

We further analyzed IPSC kinetics by selectively stimulating interneurons using dual recordings between interneurons and dLGN thalamocortical neurons. Somatically generated action potentials have been shown to propagate throughout interneuron axons (F1 terminals) and dendrites (F2 terminals), triggering GABA release onto relay neurons [29]. Current-evoked action potentials of dLGN interneurons triggered unitary evoked IPSCs (ueIPSCs) with averaged delay time of 0.5 ± 0.2 ms in the postsynaptic thalamocortical neurons (Fig 3A; n = 7). The ueIPSCs of dLGN neurons were compared with mIPSCs of VB neurons with similar amplitudes. The ueIPSCs from seven dual recordings (1–7 paired neurons) were compared with VB mIPSCs in the comparable ranges of amplitudes (Fig 3B; I: 25–35 pA; II: 35–45 pA; III: 45–55 pA; IV: 55–65 pA). The ueIPSCs from pairs 1, 3, 4, and 5 had slower rise times, longer halfwidths, and lower slopes than the comparable mIPSCs from VB neurons, which would be consistent with these dLGN neurons being F2-positive neurons (Fig 3C). By contrast, the mIPSC kinetics of pairs 2, 6, and 7 were similar to those of VB mIPSCs, suggesting those pairs have only F1 terminals. The characteristics of ueIPSCs were illustrated in Table 2. We next analyzed the decay τ of each IPSC evoked by presynaptic action potential. The distribution of the decay τ of ueIPSCs from the two subgroups of pairs (presumed F2-positive: 1,3,4,5; F2-negative: 2,6,7) had two different distributions (Fig 3D, Table 2). The presumed F-positive pairs were skewed to the right presumably through the slower kinetics of the F2 terminal outputs.

**Table 2:** The characteristics of interneuron ueIPSCs.

Pair #	Amplitude(mV)	R. Time(ms)	Halfwidth(ms)	Slope(pA/ms)	Decay τ (ms)	#
1	26.0 ± 1.6	2.3 ± 0.8	13.2 ± 4.0	9.6 ± 1.9	10.6 ± 0.5	26
2	37.7 ± 3.1	1.9 ± 0.6	10.0 ± 2.7	18.2 ± 7.9	5.8 ± 0.6	32
3	38.28 ± 2.8	2.7 ± 0.7	16.7 ± 4.9	12.1 ± 4.5	12.0 ± 1.5	11
4	35.9 ± 1.4	2.7 ± 0.7	14.1 ± 2.2	12.3 ± 2.3	9.7 ± 0.8	33
5	48.0 ± 2.3	3.0 ± 0.6	14.6 ± 3.1	13.7 ± 4.1	8.8 ± 0.5	45
6	61.9 ± 5.8	1.5 ± 0.4	8.0 ± 1.7	37.0 ± 13.3	5.3 ± 0.3	31
7	58.6 ± 2.1	2.1 ± 0.5	12.9 ± 2.8	24.9 ± 1.7	8.2 ± 0.5	41

## Discussion

In the present study, we characterized the kinetics of GABA_A_R-mediated currents originating from axonal and/or dendritic inputs onto thalamocortical neurons in visual thalamus. The GABA_A_R-mediated IPSCs displayed distinct kinetics depending on their origin: IPSCs arising from dendritic F2 outputs showed slower rise times and longer halfwidths compared to those arising from axonal F1 outputs. Such a distinct characteristic illustrates functional difference on visual information processing.

GABAergic inhibition does not only play an role in sensory information processing through thalamocortical circuits, but also in intrathalamic oscillatory activities associated with sleep/wake states and certain pathophysiological conditions such as absence epilepsy [9, 10, 12, 30]. Interneurons and TRN neurons provide inhibition to thalamocortical neurons through two distinct outputs: classical axonal outputs (F1 terminals) and presynaptic dendritic outputs (F2 terminals). In dLGN, F2 terminals are hypothesized to focally regulate retinogeniculate transmission [28]. The signaling through F2 terminals is thought to occur independent of activity at the somatic level of the interneuron and therefore provide focal inhibitory output [16, 24, 31]. In contrast to F2 terminals, it has been suggested that activation of axonal outputs (F1 terminals) of either interneurons or TRN neurons produce more of a widespread inhibition on thalamocortical neurons from their more widespread axonal arbors [28]. Somatically evoked Na^+^ dependent action potentials can trigger GABA release of axonal F1 terminals and back propagate throughout the dendritic arbor of the interneuron potentially activating F2 terminals as well, which would also lead to a global form of inhibition. Therefore, the suprathreshold somatic depolarization of interneurons would produce two forms of inhibition with distinct temporal properties: conventional F1 inhibitory and dendritic F2 inhibitory currents [29].

While there may be many factors that could impact the differences observed in ISPC kinetics, these differences may also results from different GABA_A_R subunit composition. Different combination of GABA_A_R subunits have been reported in TRN dLGN neurons and VB neurons [32, 33]. VB neurons expressed α1, α3, α5, β1, β3, γ2, γ 3, and δ subunits [32, 34, 35] while dLGN neurons had abundant expression of γ1, γ2, and δ subunits [36, 37]. It has been also established that α1 subunit-containing GABA_A_ receptors produce faster kinetics with a decay of only a few milliseconds [38], than α3 subunit-containing GABA_A_ receptors having slow responses of tens of milliseconds [39] and α6 subunit-containing GABA_A_ receptors of about hundred milliseconds [40]. VB neurons expressed a high level of slow δ subunits which are known to largely present in extrasynaptic GABA_A_ receptors. It is also known that slower IPSCs are attributed to activation of γ1 subunit-containing GABA_A_ receptors [41], while faster IPSCs were likely mediated by the γ2 subunit [42–44]. Interestingly, slow γ1-mediated currents were found in dLGN [36] which is in contrast to the fast γ2-mediated currents in VB neurons. Considering the difference in F1- and F2-terminal dependent IPSCs, we would predict that γ1 subunits may be associated with dendrodendritic synapses as opposed to axodendritic synapses. Another potential difference is that presynaptic GABA release through the activation of L-type calcium channels is responsible for slow dendritic outputs but not axonal outputs of local interneurons [29]. This is consistent with our finding of slower kinetics by F2 synapses. A similar mechanism is present at dendrodendritic synaptic transmission in olfactory periglomerular neurons [45].

GABAergic inhibition can modulate visual signals by enhancing sensitivity of contrast detection [3–5, 46]. F2 terminals in thalamus are associated with X-type neurons and these thalamocortical neurons are associated with contrast sensitivity. In this regard, our findings may delineate inhibitory mechanisms involved in contrast gain control [22, 25]. Distinct output types (F1 and F2) of interneurons found in the present study could give differential functional implication on its target cells. For example, the feed-forward inhibition driven by fast IPSC kinetics is likely optimized for time-locked temporal coding. On the other hand, slower F2-mediated PSCs can retain wide integration window, therein simultaneously modulating massive excitatory inputs especially during high frequency firing of retina ganglion cells. Consistent with this hypothesis, F2 outputs were proposed to provide prolonged inhibition onto relay neurons in activity-dependent manner [24, 29]. Likewise, the increment of contrast was mediated by enhanced response of the X-type of LGN target cells [47]. Based on these observations, we would predict that elongated inhibition of F2 outputs can contribute to heavy inhibitory tone on off-targeted excitatory relay neurons.
